# Microsatellite analysis reveals low genetic diversity in managed populations of the critically endangered gharial (*Gavialis gangeticus*) in India

**DOI:** 10.1038/s41598-021-85201-w

**Published:** 2021-03-11

**Authors:** Surya Prasad Sharma, Mirza Ghazanfarullah Ghazi, Suyash Katdare, Niladri Dasgupta, Samrat Mondol, Sandeep Kumar Gupta, Syed Ainul Hussain

**Affiliations:** Wildlife Institute of India, Chandrabani, P.O. Box # 18, Dehra Dun, Uttarakhand 248002 India

**Keywords:** Ecology, Genetics

## Abstract

The gharial (*Gavialis gangeticus*) is a critically endangered crocodylian, endemic to the Indian subcontinent. The species has experienced severe population decline during the twentieth century owing to habitat loss, poaching, and mortalities in passive fishing. Its extant populations have largely recovered through translocation programmes initiated in 1975. Understanding the genetic status of these populations is crucial for evaluating the effectiveness of the ongoing conservation efforts. This study assessed the genetic diversity, population structure, and evidence of genetic bottlenecks of the two managed populations inhabiting the Chambal and Girwa Rivers, which hold nearly 80% of the global gharial populations. We used seven polymorphic nuclear microsatellite loci and a 520 bp partial fragment of the mitochondrial control region (CR). The overall mean allelic richness (Ar) was 2.80 ± 0.40, and the observed (Ho) and expected (He) heterozygosities were 0.40 ± 0.05 and 0.39 ± 0.05, respectively. We observed low levels of genetic differentiation between populations (*F*_ST_ = 0.039, *P* < 0.05; *G’*_ST_ = 0.058, *P* < 0.05 Jost’s *D* = 0.016, *P* < 0.05). The bottleneck analysis using the *M* ratio (Chambal = 0.31 ± 0.06; Girwa = 0.41 ± 0.12) suggested the presence of a genetic bottleneck in both populations. The mitochondrial CR also showed a low level of variation, with two haplotypes observed in the Girwa population. This study highlights the low level of genetic diversity in the two largest managed gharial populations in the wild. Hence, it is recommended to assess the genetic status of extant wild and captive gharial populations for planning future translocation programmes to ensure long-term survival in the wild.

## Introduction

The order Crocodylia is represented by 27 species and three families: Alligatoridae, Crocodylidae and Gavialidae^1^. India harbours three crocodylian species: the gharial (*Gavialis gangeticus*), the mugger or marsh crocodile (*Crocodylus palustris*), and the saltwater crocodile (*Crocodylus porosus*). The gharial has the narrowest distribution range and is the most threatened species among all other crocodylian species occurring in the Indian subcontinent. Until the early twentieth century, the gharial was widely distributed in the Indus, Ganges, Mahanadi, Brahmaputra, Kaladan and Irrawaddy River systems spanning across Pakistan, India, Nepal, Bangladesh, Bhutan, and Myanmar^2–4^. Over the years, the gharial has suffered a population decline of over 80% and substantial range contraction due to habitat loss, poaching, and mortalities in passive fishing^5–7^. The species is now extinct in Myanmar, Bhutan and Pakistan. The extant gharial population is restricted to a few major river systems in India, Nepal and Bangladesh. It is presently listed as ‘critically endangered’ on the IUCN Red List of threatened species and is highly conservation dependent^7^.

In India, by the early 1970s, the gharial was restricted to few isolated locations in the Ganga, Mahanadi and Brahmaputra River systems^2^. Due to the alarming decline in the gharial populations throughout its range, the Government of India listed it in Schedule I of the Indian Wild Life (Protection) Act, 1972 to provide enhanced protection. Subsequently, conservation translocation programmes, including reintroduction and restocking, were initiated in 1975 to restore its population in the wild^5^. As a part of these programmes, eggs collected from the wild were hatched and reared in rehabilitation centres, and individuals after attaining a length of 1.2 m were translocated into suitable habitats within the newly created protected areas^5,6^.

In India, the head-start gharial conservation programme was initiated at the Chambal, Girwa and Mahanadi Rivers, where adult breeding populations existed. Initially, four facilities were established, viz. (1) Gharial Rehabilitation Centre, Tikarpada; (2) Gharial Rehabilitation Centre, Katerniaghat; (3) Gharial Rehabilitation Centre, Kukrail; and (4) Deori Gharial Rearing Centre, Morena targeting the Mahanadi, Girwa and Chambal populations. Hatchlings reared at these centres were released at the targeted rivers. In the Kukrail Centre, gharial hatchlings from 240 eggs collected from Chambal and 38 eggs collected from Girwa were reared during 1975^8,9^. Although there is no record, the hatchlings from these two rivers were likely intermixed while rearing. The hatchlings produced in Kukrail and Deori were also translocated to several other rivers, viz. Son, Ken and Ramganga, some of which have started breeding^10^. Eggs were also collected from the Narayani River in Nepal, and hatchlings were released in the Girwa river. Additionally, conservation breeding was initiated in several zoos, such as the Nandankanan Biological Park and Madras Crocodile Bank, which contributed stocks for translocation^11^. A male gharial from the Frankfurt Zoo, Germany, was obtained, which also contributed to developing stocks in Odisha for translocation^12^. All translocation sites are now protected as wildlife sanctuaries under the Indian Wild Life (Protection) Act, 1972. In the last four decades, over 5000 gharial individuals have been translocated in more than 12 rivers in India, with over 3500 individuals in the Chambal and Girwa Rivers^12^. At the beginning of the restocking programme, the gharial population in the Chambal River was 107 individuals, and now, it is approximately 1675 individuals of different ages and sexes^4,13^. The Chambal River within the National Chambal Sanctuary (425 km) has been a stronghold of the gharial population since the initiation of the translocation programme. The Girwa River originally had a small population of approximately 34 individuals of different ages and sexes^11^ and now has 43 adult individuals restricted to a 20 km stretch of the Girwa River falling within the Katerniaghat Wildlife Sanctuary. Presently, these two populations hold nearly 80% of the extant wild populations of gharial^7^.

Effective translocation programmes of endangered species essentially depend on prior knowledge of the ecological role, availability of suitable habitat, climatic requirements, behaviour, life-history traits, adequate post-release monitoring, demographic, behavioural and ecological aspects^14–16^. The effectiveness of any translocation programme is also influenced by the level of genetic diversity of the source population^17^, affecting individual and population fitness, resilience against environmental change and long-term persistence^18^. However, most translocation programmes rarely evaluate this factor^17^. Therefore, understanding the genetic status of the translocated population is vital for determining the effectiveness of translocation. Since the initiation of the gharial conservation programme, several studies have been carried out to determine their ecological features^2,4,6,13,19–21^. Nevertheless, studies on the genetic status of wild and captive gharial populations are limited^22,23^.

**Figure 1:**
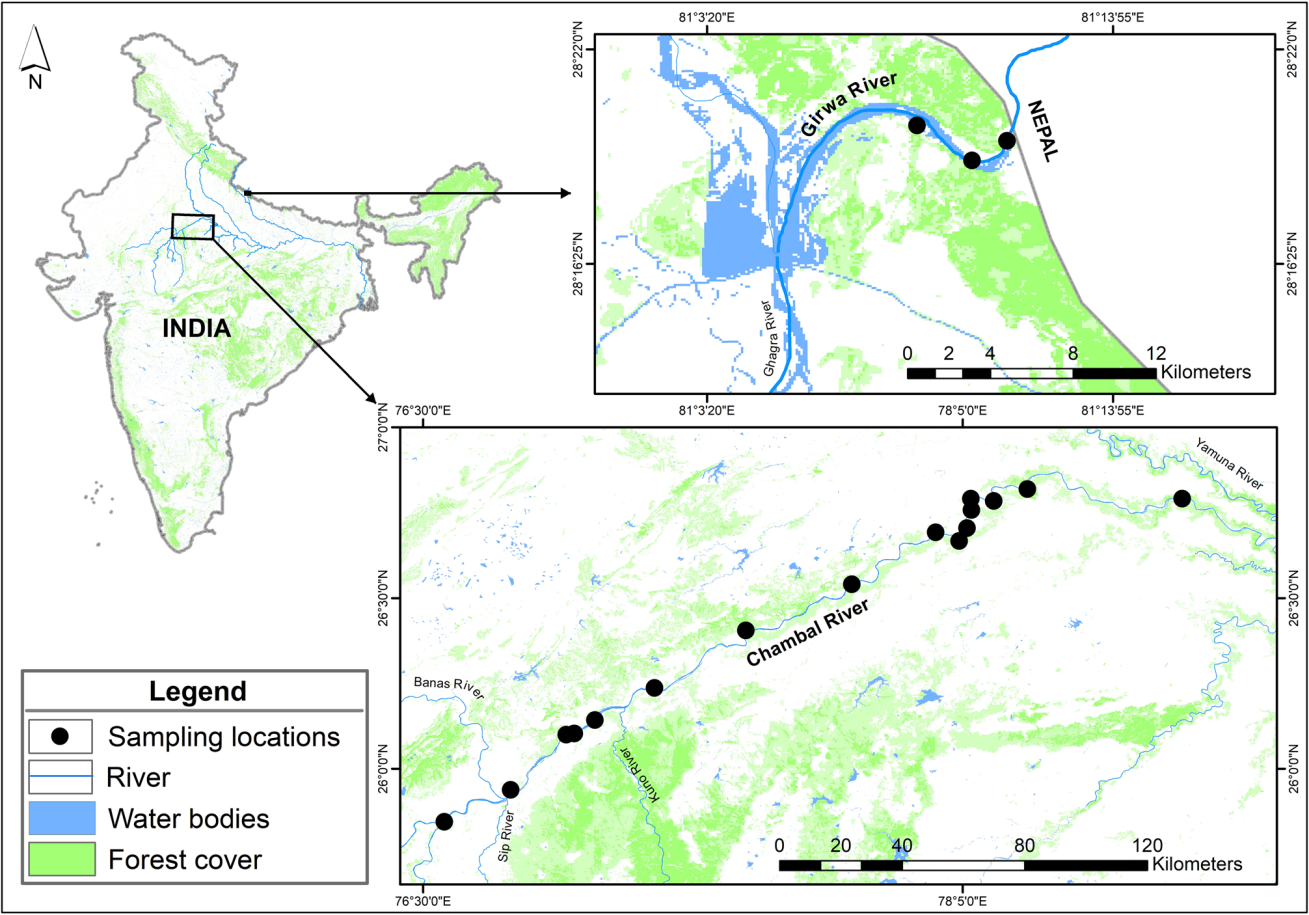
Map showing detailed representation of the study area and sampling locations along the Chambal and Girwa Rivers. The map was prepared using ArcGIS v.10.3.1 software developed by ESRI (https://www.esri.com).

In this study, we aim to assess the genetic diversity, population genetic structure and evidence of genetic bottlenecks using nuclear microsatellite loci and the mitochondrial control region (CR) of the two largest managed populations of gharials inhabiting the Chambal and Girwa Rivers (Fig. 1) to answer following research questions: (a) Do the two largest managed gharial populations differ in terms of genetic diversity? (b) Do the Chambal and Girwa River populations have their own genetic signatures? (c) If so, what is the level of differentiation? and (d) Did the demographic decline suffered by gharial populations in the last century also induce a genetic bottleneck?

## Results

### Microsatellite selection, screening and genotyping

Out of the 27 nuclear microsatellite loci (11 species-specific and 16 cross-species) screened, 18 loci showed successful amplification, and nine loci failed to amplify using known gharial samples (see Supplementary Table S1 online). We successfully genotyped 18 loci for 348 samples (Chambal = 232; Girwa = 116). Furthermore, out of 18 loci, 11 loci were found to be monomorphic and thus removed from further analyses. Overall, we used microsatellite genotype data of seven polymorphic loci from 348 gharial samples for population genetics analyses.

### Genotyping quality and error rates

The polymorphic loci average amplification success rate was 96.09% for Chambal samples and 95.38% for Girwa samples. The quality index across the seven polymorphic loci was 0.86 ± 0.01 (mean ± SE) in Chambal and 0.89 ± 0.01 in Girwa (see Supplementary Figs. S1 and S2 online). The average allelic dropout (ADO), false allele (FA) and null allele frequency across polymorphic loci were below 5% in both populations (see Supplementary Table S2 online). We could not detect the occurrence of large allele dropout in our data.

### Genetic variation

The cumulative probability of identity (PID biased) value of the panel of seven polymorphic markers was 1.74 × 10^–3^, and the probability of identity (PID sibs) was 4.84 × 10^–2^. We identified a total of 228 (Chambal = 162; Girwa = 66) distinct individuals using multilocus genotype data. The genetic diversity estimates for the Chambal and Girwa populations are summarised in Table 1. The number of alleles observed at each locus ranged from 2 to 7. The overall mean allelic richness was 2.80 ± 0.40, and the observed (Ho) and expected (He) heterozygosities were 0.40 ± 0.05 and 0.39 ± 0.05 across seven polymorphic loci, respectively. The mean allelic richness estimated using the rarefaction approach was 2.91 ± 0.60 in the Chambal population and 2.48 ± 0.48 in the Girwa population. The mean observed and expected heterozygosities were 0.42 ± 0.07 and 0.41 ± 0.06 in the Chambal and 0.42 ± 0.08 and 0.42 ± 0.09 in the Girwa populations, respectively. The inbreeding coefficient (*F*) was − 0.03 for both populations. None of the loci showed significant deviation from HWE following Bonferroni correction.

**Table 1:** Genetic diversity of 228 individuals at seven microsatellite nuclear loci.

Population	Chambal river						Girwa river					
Locus	N	Na	Ar	Ho	He	*F*	N	Na	Ar	Ho	He	*F*
G13_7	160	2	2.0	0.31	0.33	0.07	66	2	2.0	0.50	0.49	− 0.02
Cj16	158	2	2.0	0.27	0.27	− 0.01	66	2	2.0	0.03	0.03	− 0.02
G13_2	162	2	2.0	0.54	0.50	− 0.09	66	2	2.0	0.56	0.48	− 0.17
G13_5	153	3	3.0	0.24	0.23	− 0.05	Monomorphic					
G13_8	159	7	6.3	0.74	0.67	− 0.11	66	5	5.0	0.53	0.63	0.16
G13_14	160	3	3.0	0.42	0.42	0.00	66	3	3.0	0.42	0.52	0.19
G13_16	160	2	2.0	0.44	0.44	− 0.01	66	2	2.0	0.47	0.36	− 0.31
Mean		3	2.91	0.42	0.41	− 0.03		2.67	2.48	0.42	0.42	− 0.03
SE		0.69	0.60	0.07	0.06	0.02		0.49	0.48	0.08	0.09	0.08

Number of individuals sampled (N), number of alleles per locus (Na), standardised number of alleles (Ar), observed heterozygosity (Ho), expected heterozygosity (He), Inbreeding coefficient (*F*).

### Population genetic structure, differentiation and migration

**Figure 2:**
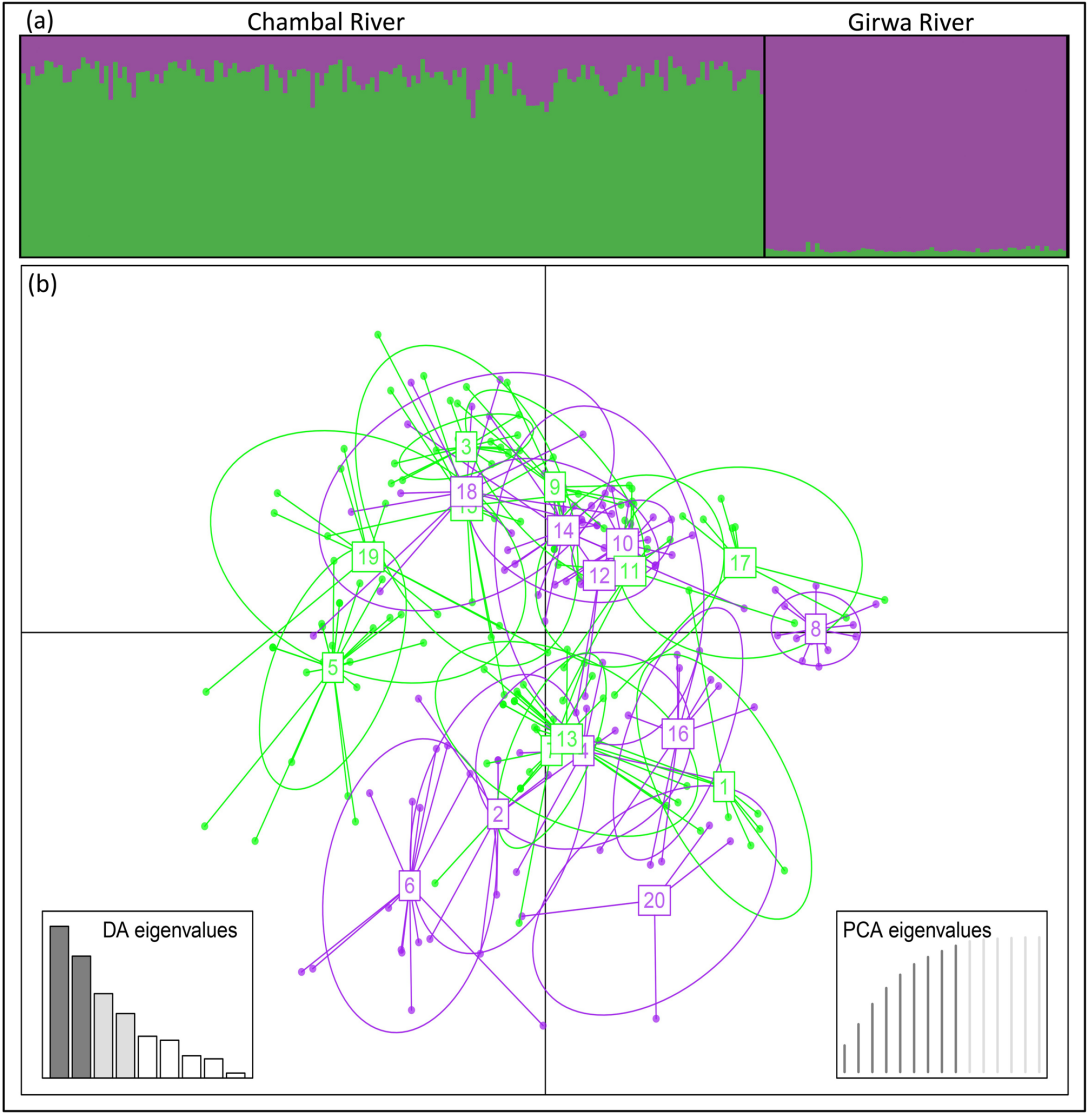
(**a**) Population genetic structure of 228 gharial individuals derived using a Bayesian clustering approach implemented in STRUCTURE. A single bar plot represents the individual, and the extent of colour in each bar indicates the probability of assigning the individual to a particular cluster. (**b**) Scatterplot of the Discriminant Analysis of Principal Components (DAPC) of 228 gharial individuals. This scatterplot shows the first two principal components of the DAPC.

The Bayesian approach implemented in Structure v2.3.4 identified two (*K* = 2) optimum number of clusters inferred by the likelihood distribution *L*(*K*) and delta *K* estimates (see Supplementary Fig. S3 online). All Girwa samples were assigned to cluster-I, with an average proportion of membership (*q*) of 0.97, and Chambal samples were assigned to cluster-II, with *q* = 0.82 (Fig. 2a). Genetic differentiation measures were low but significant as derived using both the fixation indices: *F*_ST_ = 0.039 (*P* < 0.05); *G’*_ST_ = 0.058 (*P* < 0.05) and allelic differentiation index Jost’s *D* = 0.016 (*P* < 0.05).

The discriminate analysis of principal component (DAPC) identified 20 genetic clusters associated with the lowest Bayesian information criterion (BIC) (see Supplementary Fig. S4 online). All the identified clusters (*K* = 20) showed an overlapping pattern with no clear structuring (Fig. 2b). Furthermore, the recent migration rate (*m*) was 0.12 (95% CI 0–0.26) from Chambal to Girwa River and 0.02 (95% CI 0–0.05) from Girwa to Chambal.

### Bottleneck

The *M* ratio calculated across polymorphic loci was 0.31 ± 0.06 in Chambal and 0.41 ± 0.12 in Girwa, indicating a genetic bottleneck in both populations. The heterozygosity excess test (HET) using the stepwise mutation model (SMM) (Chambal *P* = 0.28; Girwa *P* = 0.08) and the two-phase model (TPM) (Chambal *P* = 0.18; Girwa *P* = 0.08) failed to detect the signature of a genetic bottleneck. We also obtained a normal L-shaped allelic distribution for both populations.

### Mitochondrial DNA variation

We observed two mitochondrial CR haplotypes H1 in 58 individuals (92%) and H2 in five individuals (8%) with a single parsimony-informative site (at the 375-nucleotide position) in the Girwa population. Haplotype, H1 was shared by both the Girwa and Chambal populations^22^, whereas H2 was unique to the Girwa population. The haplotype diversity was (mean ± SD) 0.148 ± 0.057, and nucleotide diversity was 0.00029 ± 0.00011 in the Girwa population.

## Discussion

This study is the first comprehensive genetic assessment of the critically endangered gharial and provides crucial baseline information essential for guiding the ongoing conservation efforts. The genetic diversity observed at seven polymorphic nuclear microsatellite loci was low in the gharial populations of Chambal and Girwa. Both populations had similar mean heterozygosities, and the allelic richness (Ar) was marginally higher in the Chambal population (Table 1). The overall heterozygosities at nuclear loci in the wild (Ho = 0.40 ± 0.05; He = 0.39 ± 0.05) were lower than the previously reported estimates in captive (Ho = 0.92 ± 0.02; He 0.65 ± 0.02) gharial populations using the same set of microsatellite loci. The estimated mean allelic richness was also lower in the wild population (Chambal = 2.91 ± 0.60; Girwa = 2.48 ± 0.48) than that reported in captivity (5.5 ± 0.5)^23^. A low level of heterozygosity is not rare in wild crocodylians. Similar levels of heterozygosity have been reported in other crocodylians, including *Alligator sinensis*^24^, *Alligator mississippiensis*^25^, *Caiman yacare*^26^, *Crocodylus siamensis*^27^, *Crocodylus mindorensis*^28^, *Crocodylus moreletii*^29,30^, and *Crocodylus palustris*^31,32^, as well as in other vertebrate species that have experienced demographic bottlenecks^33^. Moreover, the Girwa population exhibits two and Chambal population exhibits a single mitochondrial CR haplotype^22^. The presence of extremely low variation at a hypervariable locus is surprising because, in other crocodylian species, the reported haplotypic diversity at mitochondrial CR is considerably higher^34–38^. The genetic diversity of a species is influenced by its geographic range, abundance, demography, and life-history traits. Narrow-ranging, less abundant and long-lived species with demographically challenged population history tend to have lower genetic diversity than widely distributed, abundant species with stable demographic history^39–41^. The low abundance, narrow distribution range, complex life-history traits, and unstable demography of the gharial may have resulted in the observed low level of genetic diversity.

The coefficient of inbreeding (*F*), primarily measures the deviation from HWE (where *F* = 0 indicates that the population is at HWE) and is often only weakly correlated with inbreeding and fitness^42,43^. The estimate is likely to be affected by several factors including use of a limited number of loci, closely related individuals, genetic bottleneck, gene flow and admixture^44–46^. Additionally, populations that experience demographic bottlenecks do not necessarily become inbred. Our estimate of *F* ≈ 0, shows that the populations are at or near HWE.

### Population genetic structure, differentiation, and migration

The presence of population genetic structure was supported by Structure (Fig. 2a) and genetic differentiation indices (*F*_ST_ = 0.039, *P* < 0.05; *G’*_ST_ = 0.058, *P* < 0.05 Jost’s *D* = 0.016, *P* < 0.05) but not by multivariate analysis DAPC (Fig. 2b). The Bayesian approach implemented in Structure is a model-based approach that largely depends on the assumption of population genetics models. In contrast, DAPC is a model-free approach that does not rely on population genetics models. DAPC performs better than Structure in characterizing population clusters^47^. Therefore, we believe that the result of DAPC is more reliable and appropriate for our dataset. The observed low level of differentiation and an admixed population structure suggested by DAPC hint towards possible intermixing of gharial individuals during the translocation programme when eggs from both the Chambal and Girwa Rivers were reared in the Kukrail centre.

Furthermore, we observed a migratory relation between the two populations. This supports our hypothesis of possible mixing of individuals during the translocation programme. It is important to note that the observed migration rate from Chambal to Girwa was higher than that from Girwa to Chambal, which is possible due to large number of eggs were sourced from Chambal and later released in the Girwa River. We could not perform genetic structure, differentiation, and migration analyses using mitochondrial CR because of low variability in the analysed region.

### Bottleneck

The estimates of the *M* ratio (Chambal = 0.31 ± 0.06; Girwa = 0.41 ± 0.12) were lower than the critical value (*M*_C_ = 0.68), confirming the signature of genetic bottlenecks in both populations. In contrast, the HET failed to detect the signature of a genetic bottleneck. This discrepancy between the results could be due to the limited statistical power of the two analyses. The *M* ratio can effectively detect genetic bottlenecks for a greater amount of time (up to 50 generations), whereas HET can efficiently detect bottleneck that occurred within a short time, 0.2–4.0 *N*e (effective population size) generations^48,49^. The gharial population has suffered two instances of range-wide population decline during the early-1970s and mid-1990s, when the population dwindled to less than 200 adult individuals in the wild^6,20^. The average generation time estimates for gharial individuals is 25 years^7^, suggested that the latter decline in population occurred very recently (approx. 1–1.5 generation ago). Hence, the undetectability of the bottleneck through HET could be due to the recent bottleneck event. Additionally, bottleneck detection can be masked due to various confounding factors including time of occurrence, duration, magnitude, gene flow, pre-bottleneck genetic variability, mating system, sample size and a limited number of loci^50–52^. Hence, there is a need to investigate both of these populations with extensive microsatellite loci to achieve high statistical power^49,53,54^.

In conclusion, low polymorphism was observed in species-specific and cross-species microsatellite loci; it remains crucial to develop novel microsatellite loci for population genetic studies. We acknowledge that our study might have been influenced by the inclusion of closely related individuals from each nesting site. However, the sampling was conducted from a large number of nests spread across 19 nesting sites. Therefore, we believe that our sampling strategy potentially represents the level of genetic diversity of the studied populations and minimises the bias arising from closely related individuals.

### Conservation implication

Gharial translocation is considered as one of the most successful species recovery programmes in the world^12^ in terms of increasing the abundance of the species in the wild. However, despite a long history of conservation efforts, limited information on the genetic status of the gharial has hindered, if not precluded, effective conservation planning. Gharial populations inhabit different biogeographic zones of India and are adapted to specific environmental conditions, yet translocation programmes have not considered the effects of genetic intermixing among populations. Such intermixing of populations without prior knowledge of the genetic structure can potentially threaten the genetic integrity and distinctiveness of the resident populations^14^. Our study augments the existing knowledge on the genetic status of the critically endangered gharial, which is vital for future translocations and research. This study highlighted low levels of genetic diversity and admixed structure in the two largest managed populations of gharial in the wild, which are considerably lower than the previously reported estimates of captive populations^23^.

Moreover, the gharial is a highly conservation-dependent species, and translocations without assessing the genetic status of the source populations may further deteriorate the level of genetic diversity in the translocated populations. Hence, we recommend limiting the interpopulation release of individuals to prevent further intermixing of the gene pool until information on the genetic status of the extant wild and captive gharial populations using an extensive dataset of mitochondrial and microsatellite markers is available. Information on the genetic status will assist in the identification of potential source populations and maintain adequate levels of genetic diversity to secure the continuing persistence of gharials in the wild.

## Methods

### Research permits and ethical considerations

The Forest Department of Madhya Pradesh (Letter No. SL/Tech-1/8200), Rajasthan (Letter No. 1399) and Uttar Pradesh (Letter No. 3093 and 3778(A)) provided all required permissions for the survey and collection of the biological samples. As all samples were collected from either dead remains of hatchlings or hatched eggshells, no animal ethical clearance was required for this study.

### Sample collection and DNA extraction

Sampling of a large number of adult individuals required for population genetic assessments has its own ethical and logistical constraints, especially when the species in question is critically endangered. Considering these restrictions, we collected tissue from the remains of dead hatchlings and chorioallantoic membranes from hatched eggshells shortly after hatching. Samples were collected from 16 nesting sites along the Chambal River and three nesting sites along the Girwa River during 2017 and 2018, respectively (Fig. 1). We collected 348 samples (Chambal = 232 samples and Girwa = 116 samples) for genetic assessment (see Supplementary Table S3 online). Out of 348 samples, 49 samples were unique (one sample per clutch), 138 samples were from a sibling (more than one sample per clutch), and 161 samples had no clutch information (see Supplementary Table S4 online). All samples were stored in absolute ethanol at room temperature and later at − 20 °C in the laboratory for long-term storage. Total genomic DNA was extracted from tissue (64 ± 19 mg) (mean ± SD) and chorioallantoic membrane using the phenol–chloroform method^55^. Cotton swabs were used when chorioallantoic membrane was unable to separate from eggshells.

### Microsatellite selection, screening and genotyping

We initially screened 11 of the 18 gharial specific microsatellite loci described by Jogayya et al.^23^. However, only six of the eleven loci were found to be polymorphic. Hence, we conducted an exhaustive literature survey to identify the potential cross-species loci used across families Alligatoridae, Crocodylidae and Gavialidae. We found a total of 424 previously described microsatellite loci and listed the loci based on successful cross-species transferability, allele diversity, heterozygosity, polymorphic information content and allele range. Finally, we selected 16 cross-species loci developed in eight different crocodylian species for screening. Out of 16, a total of 12 loci were selected based on successful cross-species transferability in other crocodilian species. The remaining four loci were selected based on a number of alleles ≥ 4, Ho and He ≥ 5, PIC ≥ 0.5 and allelic range < 300 bp. The list of microsatellite loci screened in the current study is provided in Supplementary Table S1 online.

Polymerase chain reactions (PCR) were performed in 10 μL reaction volumes containing 5 μL of 2 × QIAGEN Multiplex PCR master mix, 2 μL of 5 × Q-solution (QIAGEN Inc., Germany), and the labeled forward primer at 0.15 μM and the reverse primer at 0.15 μM and 2 μL of DNA template. The thermal profiles included an initial denaturation at 95 °C for 15 min, followed by 35 cycles at 94 °C for 40 s, Ta at 56–62 °C (see Supplementary Table S1 online) for 60 s, 72 °C for 60 s and a final extension of 72 °C for 30 min. The amplified products were genotyped using the GeneScan 500 LIZ dye size standard (Applied Biosystems) in 3500XL Genetic Analyzer (Applied Biosystems). The alleles were scored using the program GeneMarker v2.7.4 (SoftGenetics, LLC) with combined automated allele scoring and validated through visual inspection. Three replicates of each sample were carried out to obtain reliable multilocus genotypes using noninvasive samples following a multiple-tube approach^56^.

### Mitochondrial CR DNA sequencing

We selected a 520 bp partial fragment mitochondrial CR to assess the genetic variation in gharials. We used primers (L15637 5′-GCATAACACTGAAAATGTTAAYATGG-3′ and H16258 5′-CTAAAATTACAGAAAAGCCGACCC-3′) described by Oaks (2011) to amplify the selected fragment^57^. The PCRs were carried out in 20 μL volumes containing 2 μL of the DNA template, 10 μL of 2 × PCR buffer, 0.2 mM of dNTPs, 0.25 μL of each primer, and 0.1 μL (0.5 units) of DreamTaq DNA polymerase (Thermo Scientific). The thermal profile was 95 °C for 5 min, followed by 35 cycles at 95 °C for 35 s, 56 °C for 40 s, 72 °C for 45 s, and a final extension of 72 °C for 10 min. The amplified products were visualised using a 2% agarose gel. The positive amplicons were cleaned up with Exonuclease-I (Thermo Scientific) and Shrimp Alkaline Phosphatase (Applied Biosystems), and sequenced using forward primers in 3500XL Genetic Analyzer (Applied Biosystems).

### Data analysis

### Genotyping quality and error rates

We obtained a consensus genotype and estimated the quality index for each locus genotyped following Miquel et al.^56^. We scored each repeat with ‘1’ if the genotype was identical to the consensus and ‘0’ if the genotype did not match the consensus due to any errors such as no amplification, allelic dropout, or false allele^56^. We estimated the average amplification success as a percent of positive PCR amplification. We estimated the frequency of genotyping errors due to allelic dropout (ADO) and false allele (FA) following Broquet et al.^58^. The frequency of null alleles was estimated using FreeNa^59^ and occurrences of large allelic dropout using Micro-Checker v2.2.3^60^.

We used samples with a quality index above 0.67 per locus (identical genotypes in two replicates out of three replicates) and a mean quality index across loci above 0.75 for analyses.

### Genetic variation

We calculated the probability of identity PID (biased) and PID (sibs) using Gimlet v1.3.3^61^. We estimated summary statistics (number of alleles per locus, observed heterozygosity, expected heterozygosity, and inbreeding coefficient) using GenAlEx v6.0^62^ and deviations from Hardy–Weinberg equilibrium (HWE) for each locus using Bonferroni correction in Cervus v3.0.7^63^. We also estimated allelic richness using a rarefaction approach implemented in HP-Rare v1.1 to account for the uneven sample size of the two populations^64^.

### Population structure, differentiation, and migration

We have estimated the genetic differentiation using fixation indices *F*_ST_^65^_,_ *G’*_ST_^66^ and allelic differentiation index Jost’s *D*^67^ using the *strataG* package^68^ implemented in R studio. The estimates were calculated using 10^3^ bootstrap iterations. We inferred the population genetic structure using the Bayesian approach implemented in Structure v2.3.4^69^. Structure is a systematic model-based Bayesian clustering approach that uses allele frequencies at each locus to infer the population structure. The analysis was performed for 1–10 clusters (*K*). For each *K,* ten iterations were run under the admixture model with correlated allele frequencies and sampling location as a priori. The LOCPRIOR (sampling location as a priori) model performs well when no clear signal of a structure is detected or when there is low genetic differentiation, limited loci or a limited sample size^70^. The simulations were run for 10^5^ burn-in and 10^6^ Markov chain Monte Carlo iterations (MCMC). The optimum number of *K* was inferred using the likelihood distribution *L*(*K*) and the delta *K*^71^, which were estimated using the web version of Structure Harvester, v0.6.94^72^. The assignment plot was prepared using the program Distruct v1.1^73^. Additionally, we used the DAPC, a multivariate nonmodel-based approach, to identify and describe genetic clusters^47^. The optimal number of the clusters was estimated based on the lowest associated BIC. The analysis was performed using the *adgenet* in R studio.

We used BayesAss v3.0 to estimate the recent migration rate (*m*) between Chambal and Girwa populations^74^. The simulations (n = 3) were run with different seed numbers using 10^7^ MCMC iterations and 10^6^ burn-in periods.

### Bottleneck detection

We estimated demographic changes using two qualitative approaches: (a) the Garza-Williamson index (or *M* ratio) implemented in Arlequin v3.1^75^ and (b) the HET approach implemented in Bottleneck v1.2.02^76^. The *M* ratio estimates the ratio of the observed number of alleles to the size of allele range based on the assumption that in a recently reduced population, the ratio is expected to decrease due to random loss of alleles in a population. The calculated *M* ratio was then compared with the critical value (*M*_C_ = 0.68). An *M* ratio below *M*_C_ is considered as a signature of genetic bottleneck^77^. The HET approach assumes that in a recently reduced population, an excess of the gene diversity under Hardy–Weinberg equilibrium is expected relative to gene diversity under mutation-drift equilibrium. We used one-tailed Wilcoxon test to determine the presence of a significant number of loci with excess heterozygosity. The estimates were calculated under two mutation models: the SMM and TPM. The TPM tends to be the most appropriate mutation model for microsatellite loci^78^. The TPM was carried out at 95% SMM (variance at 12), and the simulations were run for 10^4^ iterations^76^.

### Mitochondrial DNA variation

We generated 63 mitochondrial CR sequences from the Girwa population and submitted it to GenBank (Accession No. MT500792–MT500854). We also obtained previously published mitochondrial CR sequences of the Chambal population (n = 103) from GenBank (Accession No. MT458816–MT458918)^22^. The sequences were aligned using the ClustalW algorithm^79^ in BioEdit v7.2.6^80^. The summary statistics, including the number of haplotypes, haplotype and nucleotide diversity, were estimated using DnaSP v5.10.01^81^.

## Supplementary Information

Supplementary Information.

## Data Availability

The mitochondrial sequences used in this study have been submitted to GenBank (Accession Nos. MT500792–MT500854). The microsatellite genotyping data used in the study is available on request from the corresponding author.

## References

[1] Grigg G, Kirshner D (2015). Biology and Evolution of Crocodylians.

[2] Singh, L. A. K. Ecological studies on the Indian gharial *Gavialis gangeticus* (Gmelin) (Reptilia, Crocodilia). PhD Thesis, *Utkal University*, *Odisha* (1978).

[3] Whitaker R, Webb GJW, Manolis SC, Whitehead PJ (1987). The management of crocodilians in India. Wildlife Management; Crocodiles and Alligators.

[4] Hussain SA (1999). Reproductive success, hatchling survival and rate of increase of gharial *Gavialis gangeticus* in National Chambal Sanctuary, India. Biol. Conserv..

[5] Bustard HR (1975). A future for the Gharial. Cheetal.

[6] Hussain SA (2009). Basking site and water depth selection by gharial *Gavialis gangeticus* Gmelin 1789 (Crocodylia, Reptilia) in National Chambal Sanctuary, India and its implication for river conservation. Aquat. Conserv. Mar. Freshw. Ecosyst..

[7] Lang, J. W., Chowfin, S. & Ross, J. P. *Gavialis gangeticus* (errata version published in 2019). *IUCN Red List Threat. Species 2019* (2019).

[8] Basu D (1981). Saving the gharial. Indian Wildlifer.

[9] Singh VB (1978). The status of the gharial (*Gavialis gangeticus*) in U.P. and its rehabilitation. J. Bombay Nat. Hist. Soc..

[10] Stevenson C, Whitaker R, Manolis SC, Stevenson C (2010). Indian Gharial *Gavialis gangeticus*. Crocodiles. Status Survey and Conservation Action Plan.

[11] Whitaker R, Basu D (1982). The gharial (*Gavialis gangeticus*) a review. J. Bombay Nat. Hist. Soc..

[12] Whitaker R (2007). The gharial: Going extinct again. Iguana.

[13] Lang JW, Jailabdeen A, Kumar P (2018). Gharial ecology project—Update 2018–2019. IUCN-SSC Crocodile Spec. Gr. Newsl..

[14] IUCN/SSC. Guidelines for Reintroductions and Other Conservation Translocations IUCN. *Version 1.0. Gland, Switzerland: IUCN Species Survival Commission* viiii + 57 pp. (2013).

[15] Schwartz, M. K. Guidelines on the use of molecular genetics in reintroduction programs. *EU LIFE-Nature Proj. to Guidel. reintroduction Threat. species* 51–58 (2005).

[16] White LC, Moseby KE, Thomson VA, Donnellan SC, Austin JJ (2018). Long-term genetic consequences of mammal reintroductions into an Australian conservation reserve. Biol. Conserv..

[17] Weeks AR (2011). Assessing the benefits and risks of translocations in changing environments: A genetic perspective. Evol. Appl..

[18] Hughes AR, Inouye BD, Johnson MTJ, Underwood N, Vellend M (2008). Ecological consequences of genetic diversity. Ecol. Lett..

[19] Katdare S (2011). Gharial (*Gavialis gangeticus*) populations and human influences on habitat on the River Chambal, India. Aquat. Conserv. Mar. Freshw. Ecosyst..

[20] Nair T, Thorbjarnarson JB, Aust P, Krishnaswamy J (2012). Rigorous gharial population estimation in the Chambal: Implications for conservation and management of a globally threatened crocodilian. J. Appl. Ecol..

[21] Hussain, S. A. Ecology of gharial (*Gavialis gangeticus*) in National Chambal Sanctuary. MPhil Thesis, *Aligarh Muslim University*, *Uttar Pradesh* (1991).

[22] Sharma SP (2020). Mitochondrial DNA analysis reveals extremely low genetic diversity in a managed population of the Critically Endangered Gharial (*Gavialis gangeticus*, Gmelin 1789). Herpetol. J..

[23] Jogayya KN, Meganathan PR, Dubey B, Haque I (2013). Novel microsatellite DNA markers for Indian Gharial (*Gavialis gangeticus*). Conserv. Genet. Resour..

[24] Zhu H, Wu X, Xue H, Wei L, Hu Y (2009). Isolation of polymorphic microsatellite loci from the Chinease alligator (*Alligator sinensis*). Mol. Ecol. Resour..

[25] Glenn TC (1998). Characterization of microsatellite DNA loci in American alligators. Copeia.

[26] Ojeda GN, Amavet PS, Rueda EC, Siroski PA, Larriera A (2017). Mating system of *Caiman yacare* (Reptilia: Alligatoridae) described from microsatellite genotypes. J. Hered..

[27] Yu D (2011). Analysis of genetic variation and bottleneck in a captive population of Siamese crocodile using novel microsatellite loci. Conserv. Genet. Resour..

[28] Hinlo MRP (2014). Population genetics implications for the conservation of the Philippine Crocodile *Crocodylus mindorensis* Schmidt, 1935 (Crocodylia: Crocodylidae). J. Threat. Taxa.

[29] Mcvay JD (2008). Evidence of multiple paternity in Morelet’s Crocodile (*Crocodylus moreletii*) in Belize, CA, inferred from microsatellite markers. J. Exp. Zool. Part A Ecol. Genet. Physiol..

[30] Dever JA, Strauss RE, Rainwater TR, McMurry ST, Densmore ILD (2002). Genetic diversity, population subdivision, and gene flow in Morelet’s crocodile (*Crocodylus moreletii*) from Belize, Central America. Copeia.

[31] Aggarwal RK, Lalremruata A, Dubey B (2014). Development of fourteen novel microsatellite markers of *Crocodylus palustris*, the Indian mugger, and their cross-species transferability in ten other crocodilians. Conserv. Genet. Resour..

[32] Campos JC, Mobaraki A, Abtin E, Godinho R, Brito JC (2018). Preliminary assessment of genetic diversity and population connectivity of the Mugger Crocodile in Iran. Amphib. Reptil..

[33] Garner A, Rachlow JL, Hicks JF (2005). Patterns of genetic diversity and its loss in mammalian populations. Conserv. Biol..

[34] Rossi NA (2020). High levels of population genetic differentiation in the American crocodile (*Crocodylus acutus*). PLoS ONE.

[35] van Asch B (2019). Phylogeography, genetic diversity, and population structure of Nile crocodile populations at the fringes of the southern African distribution. PLoS ONE.

[36] Luck NL (2012). Mitochondrial DNA analyses of the saltwater crocodile (*Crocodylus porosus*) from the Northern Territory of Australia. Aust. J. Zool..

[37] Russello MA, Brazaitis P, Gratten J, Watkins-Colwell GJ, Caccone A (2007). Molecular assessment of the genetic integrity, distinctiveness and phylogeographic context of the Saltwater crocodile (*Crocodylus porosus*) on Palau. Conserv. Genet..

[38] Ray DA (2004). Low levels of nucleotide diversity in *Crocodylus moreletii*and evidence of hybridization with *C. acutus*. Conserv. Genet..

[39] Eckert CG, Samis KE, Lougheed SC (2008). Genetic variation across species’ geographical ranges: The central-marginal hypothesis and beyond. Mol. Ecol..

[40] Ellegren H, Galtier N (2016). Determinants of genetic diversity. Nat. Rev. Genet..

[41] Romiguier J (2014). Comparative population genomics in animals uncovers the determinants of genetic diversity. Nature.

[42] Allendorf FW, Luikart G (2007). Conservation and the Genetics of Populations.

[43] Guries, R. P. & Ledig, F. T. Genetic structure of populations and differentiation in forest trees. in *Conkle, MT (tech. coord.) Proceedings of the symposium on isozymes of North American forest trees and forest insects. USDA For. Serv. Gen. Tech. Rep. PSW-48* 42–47 (1979).

[44] Biebach I, Keller LF (2010). Inbreeding in reintroduced populations: The effects of early reintroduction history and contemporary processes. Conserv. Genet..

[45] Wang J (2017). Estimating pairwise relatedness in a small sample of individuals. Heredity (Edinb)..

[46] Degiorgio M, Rosenberg NA (2008). An unbiased estimator of gene diversity in samples containing related individuals p. Mol. Biol. Evol..

[47] Jombart T, Devillard S, Balloux F (2010). Discriminant analysis of principal components: A new method for the analysis of genetically structured populations. BMC Genet..

[48] Girod C, Vitalis R, Leblois R, Fréville H (2011). Inferring population decline and expansion from microsatellite data: A simulation-based evaluation of the msvar method. Genetics.

[49] Luikart G, Cornuet JM (1998). Empirical evaluation of a test for identifying recently bottlenecked populations from allele frequency data. Conserv. Biol..

[50] Keller LF (2001). Immigration and the ephemerality of a natural population bottleneck: Evidence from molecular markers. Proc. R Soc. London. Ser. B Biol. Sci..

[51] Cristescu R, Sherwin WB, Handasyde K, Cahill V, Cooper DW (2010). Detecting bottlenecks using BOTTLENECK 1.2.02 in wild populations: The importance of the microsatellite structure. Conserv. Genet..

[52] Peery MZ (2012). Reliability of genetic bottleneck tests for detecting recent population declines. Mol. Ecol..

[53] Hoban SM, Gaggiotti OE, Bertorelle G (2013). The number of markers and samples needed for detecting bottlenecks under realistic scenarios, with and without recovery: A simulation-based study. Mol. Ecol..

[54] Cornuet JM, Luikart G (1996). Description and power analysis of two tests for detecting recent population bottlenecks from allele frequency data. Genetics.

[55] Sambrook J, Fritsch EF, Maniatis T (1989). Molecular Cloning: A Laboratory Manual.

[56] Miquel C (2006). Quality indexes to assess the reliability of genotypes in studies using noninvasive sampling and multiple-tube approach. Mol. Ecol. Notes.

[57] Oaks JR (2011). A time-calibrated species tree of Crocodylia reveals a recent radiation of the true crocodiles. Evolution (N.Y.).

[58] Broquet T, Petit E (2004). Quantifying genotyping errors in noninvasive population genetics. Mol. Ecol..

[59] Chapuis MP, Estoup A (2007). Microsatellite null alleles and estimation of population differentiation. Mol. Biol. Evol..

[60] Van Oosterhout C, Hutchinson WF, Wills DPM, Shipley P (2004). MICRO-CHECKER: Software for identifying and correcting genotyping errors in microsatellite data. Mol. Ecol. Notes.

[61] Valière N (2002). GIMLET: A computer program for analysing genetic individual identification data. Mol. Ecol. Notes.

[62] Peakall R, Smouse PE (2012). GenALEx 6.5: Genetic analysis in Excel. Population genetic software for teaching and research-an update. Bioinformatics.

[63] Kalinowski ST, Taper ML, Marshall TC (2007). Revising how the computer program cervus accommodates genotyping error increases success in paternity assignment. Mol. Ecol..

[64] Kalinowski ST (2005). HP-RARE 1.0—A computer program for performing rarefaction on measures of allelic richness.pdf. Mol. Ecol. Notes.

[65] Weir BS, Cockerham C (1984). Estimating F-statistics for the analysis of population structure. Evolution (N. Y.)..

[66] Hedrick PW (2005). A standardized genetic differentiation measure. Evolution (N. Y.)..

[67] Jost L (2008). GST and its relatives do not measure differentiation. Mol. Ecol..

[68] Archer FI, Adams PE, Schneiders BB (2017). stratag: An r package for manipulating, summarizing and analysing population genetic data. Mol. Ecol. Resour..

[69] Pritchard JK, Stephens M, Donnelly P (2000). Inference of population structure using multilocus genotype data: Dominant markers and null alleles. Genetics.

[70] Hubisz MJ, Falush D, Stephens M, Pritchard JK (2009). Inferring weak population structure with the assistance of sample group information. Mol. Ecol. Resour..

[71] Evanno G, Regnaut S, Goudet J (2005). Detecting the number of clusters of individuals using the software STRUCTURE: A simulation study. Mol. Ecol..

[72] Earl DA, VonHoldt BM (2012). STRUCTURE HARVESTER: A website and program for visualizing STRUCTURE output and implementing the Evanno method. Conserv. Genet. Resour..

[73] Rosenberg NA (2004). DISTRUCT: A program for the graphical display of population structure. Mol. Ecol. Notes.

[74] Wilson GA, Rannala B (2003). Bayesian inference of recent migration rates using multilocus genotypes. Genetics.

[75] Excoffier L, Laval G, Schneider S (2005). Arlequin (version 3.0): An integrated software package for population genetics data analysis. Evol. Bioinforma. Online..

[76] Piry S, Luikart G, Cornuet JM (1999). BOTTLENECK: A computer program for detecting recent reductions in the effective population size using allele frequency data. J. Hered..

[77] Garza JC, Williamson EG (2001). Detection of reduction in population size using data from microsatellite loci. Mol. Ecol..

[78] Di Rienzo A (1994). Mutational processes of simple-sequence repeat loci in human populations. Proc. Natl. Acad. Sci. USA.

[79] Thompson JD, Higgins DG, Gibson TJ (1994). CLUSTAL W: Improving the sensitivity of progressive multiple sequence alignment through sequence weighting, position-specific gap penalties and weight matrix choice. Nucleic Acids Res..

[80] Hall TA (1999). BioEdit: A user-friendly biological sequence alignment editor and analysis program for Windows 95/98/NT. Nucleic Acid Symp. Ser..

[81] Librado P, Rozas J (2009). DnaSP v5: A software for comprehensive analysis of DNA polymorphism data. Bioinformatics.

