# Supplementary material for: Microsatellite analysis reveals low genetic diversity in managed populations of the critically endangered gharial (Gavialis gangeticus) in India
Source: Sci Rep. 2021 Mar 11;11:5627. doi: 10.1038/s41598-021-85201-w (PMC7970970; doi:10.1038/s41598-021-85201-w)
Supplement: Supplementary file 1 — Supplementary Information. [file 41598_2021_85201_MOESM1_ESM.docx]

**Microsatellite analysis reveals low genetic diversity in managed populations of the critically endangered gharial (*Gavialis gangeticus*) in India**

Surya Prasad Sharma^1^, Mirza Ghazanfarullah Ghazi^1^, Suyash Katdare^1^, Niladri Dasgupta^1^, Samrat Mondol^1^, Sandeep Kumar Gupta^1^ and Syed Ainul Hussain^1*^

^1^ Wildlife Institute of India, P.O. Box # 18, Chandrabani, Dehra Dun, 248002, Uttarakhand, India.

***Corresponding author**- Syed Ainul Hussain, E-mail: hussain@wii.gov.in

**Table S1**: List of microsatellite markers screened for population genetic study**.** * No amplification.

| **Locus** | **Repeats motif** | **Number of alleles** | **Allele Range(bp)** | **T_a_ (⁰C)** | **Species** | **Reference** |
| --- | --- | --- | --- | --- | --- | --- |
| G13_8 | tetra | 8 | 330-382 | 60 | *Gavialis gangeticus* | [1] |
| G13_5 | tetra | 3 | 256-264 | 56 | *Gavialis gangeticus* | [1] |
| G13_14 | tetra-di-tetra | 3 | 308-312 | 60 | *Gavialis gangeticus* | [1] |
| G13_2 | tetra | 2 | 290-294 | 60 | *Gavialis gangeticus* | [1] |
| G13_7 | tetra | 2 | 200-204 | 60 | *Gavialis gangeticus* | [1] |
| G13_16 | tetra-di | 2 | 255-265 | 60 | *Gavialis gangeticus* | [1] |
| Cj16 | di | 2 | 153-165 | 60 | *Crocodylus johnstoni* | [2] |
| G13_1 | tri-di | 1 | -- | 60 | *Gavialis gangeticus* | [1] |
| G13_6 | tetra | 1 | -- | 60 | *Gavialis gangeticus* | [1] |
| G13_11 | tetra-di | 1 | -- | 56 | *Gavialis gangeticus* | [1] |
| G13_15 | tetra | 1 | -- | 56 | *Gavialis gangeticus* | [1] |
| G13_18 | di-di | 1 | -- | 60 | *Gavialis gangeticus* | [1] |
| CpSSR12 | di | 1 | -- | 60 | *Crocodylus palustris* | [3] |
| 4HDZ35 | di | 1 | -- | 60 | *Crocodylus mindorensis* | [4] |
| CUD68 | di | 1 | -- | 62 | *Crocodylus acutus* | [5] |
| 4HDZ27 | di | 1 | -- | 60 | *Crocodylus mindorensis* | [4] |
| Cp309 | tetra | 1 | -- | 56 | *Crocodylus porosus* | [5] |
| TGE2 | di | 1 | -- | 56 | *Alligator sinensis* | [6] |
| CpSSR13* | di | -- | -- | -- | *Crocodylus palustris* | [3] |
| CpSSR14* | di | -- | -- | -- | *Crocodylus palustris* | [3] |
| CpP107* | tetra | -- | -- | -- | *Crocodylus porosus* | [5] |
| CpP121* | tetra | -- | -- | -- | *Crocodylus porosus* | [5] |
| CpP1401* | tetra | -- | -- | -- | *Crocodylus porosus* | [5] |
| 4HDZ391* | di | -- | -- | -- | *Crocodylus mindorensis* | [4] |
| Amiu-102* | di | -- | -- | -- | *Alligator mississippiensis* | [7] |
| Amiu-202* | di | -- | -- | -- | *Alligator mississippiensis* | [8] |
| CpP1306* | tetra | -- | -- | -- | *Crocodylus porosus* | [5] |

**Table S2:** Amplification success and error rates calculated for polymorphic loci. Amplification Success (AS); Quality Index per locus (QI); Allele Drop Out (ADO); False Allele (FA) and Null Allele (NA).

| **Populations** | **Chambal River** | | | | | **Girwa River** | | | | |
| --- | --- | --- | --- | --- | --- | --- | --- | --- | --- | --- |
| **Locus** | **AS (%)** | **QI** | **ADO** | **FA** | **NA** | **AS (%)** | **QI** | **ADO** | **FA** | **NA** |
| G13_7 | 93.65 | 0.88 | 0.07 | 0.02 | 0.02 | 98.86 | 0.88 | 0.11 | 0.03 | 0.00 |
| Cj16 | 93.53 | 0.89 | 0.02 | 0.02 | 0.00 | 84.86 | 0.85 | 0.00 | 0.00 | 0.00 |
| G13_2 | 97.63 | 0.90 | 0.03 | 0.03 | 0.00 | 95.73 | 0.91 | 0.03 | 0.03 | 0.00 |
| G13_5 | 97.45 | 0.83 | 0.06 | 0.04 | 0.00 | Monomorphic | | | | |
| G13_8 | 95.87 | 0.82 | 0.02 | 0.10 | 0.00 | 97.14 | 0.93 | 0.01 | 0.03 | 0.05 |
| G13_14 | 95.90 | 0.85 | 0.04 | 0.04 | 0.02 | 97.13 | 0.92 | 0.03 | 0.04 | 0.05 |
| G13_16 | 98.62 | 0.86 | 0.06 | 0.03 | 0.00 | 98.56 | 0.87 | 0.08 | 0.09 | 0.00 |
| **Mean** | **96.09** | **0.86** | **0.04** | **0.04** | **0.01** | **95.38** | **0.89** | **0.04** | **0.04** | **0.02** |
| **SE** | **0.74** | **0.01** | **0.01** | **0.01** | **0.004** | **2.15** | **0.01** | **0.02** | **0.01** | **0.009** |

**Table S3:** The table information on nesting site, geographic coordinates and number of samples collected from each nesting site.

| **Population** | **Nesting site** | **Latitude** | **Longitude** | **Number of samples** | **Number of unique individual(s)** |
| --- | --- | --- | --- | --- | --- |
| Chambal | Baroli | 26.10360 | 76.94320 | 44 | 32 |
| Chambal | Dangbasai | 26.54073 | 77.75784 | 41 | 26 |
| Chambal | Pali | 25.84446 | 76.56175 | 29 | 21 |
| Chambal | Jaitpura | 26.79163 | 78.10700 | 8 | 6 |
| Chambal | Kuthiyana | 26.70554 | 78.09555 | 17 | 11 |
| Chambal | Barsala | 26.78505 | 78.17372 | 12 | 9 |
| Chambal | Pureni | 26.75853 | 78.10860 | 6 | 5 |
| Chambal | Shankarpura | 26.66739 | 78.07249 | 7 | 5 |
| Chambal | Nadigaon | 26.14282 | 77.00317 | 27 | 16 |
| Chambal | Din-ka-pura | 26.79148 | 78.72742 | 2 | 1 |
| Chambal | Tigri | 26.69270 | 78.00359 | 15 | 14 |
| Chambal | Rijentha | 26.23728 | 77.17841 | 2 | 1 |
| Chambal | Bagadia | 26.75853 | 78.10860 | 4 | 4 |
| Chambal | Bharrah | 26.40531 | 77.44702 | 4 | 4 |
| Chambal | Radi | 26.23728 | 77.17841 | 4 | 1 |
| Chambal | Reha | 26.82004 | 78.27239 | 10 | 6 |
| Girwa | Pathrahna | 28.32706 | 81.18586 | 8 | 5 |
| Girwa | Ambah | 28.31850 | 81.17061 | 8 | 7 |
| Girwa | Bhawanipur | 28.33372 | 81.14678 | 100 | 54 |
| **Total** | | | | **348** | **228** |

**Table S4:** The table contains information regarding the type and origin of samples used in the current study. Unique: one sample per clutch; Sibling: more than one samples per clutch and Unknown: if the clutch information is not known.

| **Population** | **Chambal river** | | | **Girwa river** | | |
| --- | --- | --- | --- | --- | --- | --- |
| **Sample Origin** | **Total sample size** | **Tissue**  **(tail scutes)** | **Hatchling chorioallantoic membranes** | **Total sample size** | **Tissue**  **(tail scutes)** | **Hatchling chorioallantoic membranes** |
| Unique | 49 | 15 | 34 | -- | -- | -- |
| Siblings | 56 | 3 | 53 | 82 | 07 | 75 |
| No clutch information | 127 | 47 | 80 | 34 | 04 | 30 |
| **Total** | **232** | **65** | **167** | **116** | **11** | **105** |

**Fig. S1:** Quality index per sample for the Girwa river. The samples with mean quality index ≥ 0.75 were used for analysis.

**Fig. S2**: Quality index per sample for the Chambal river. The samples with mean quality index ≥ 0.75 were used for analysis.


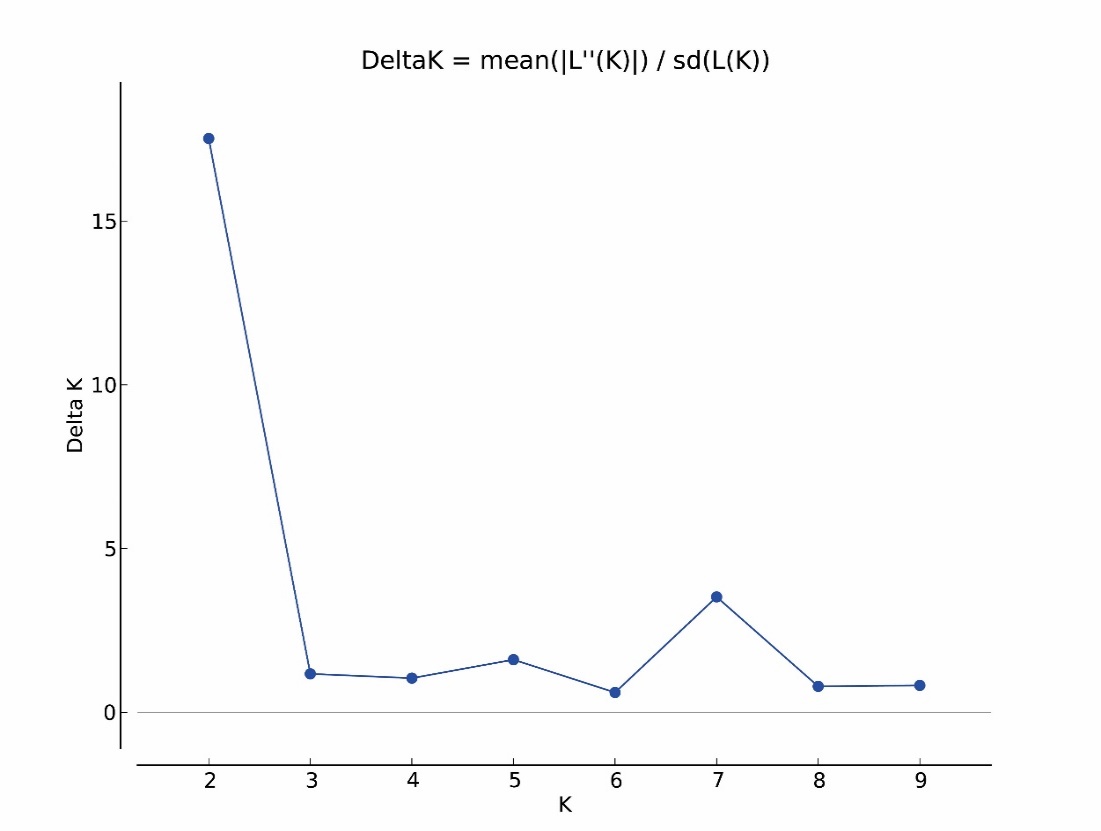

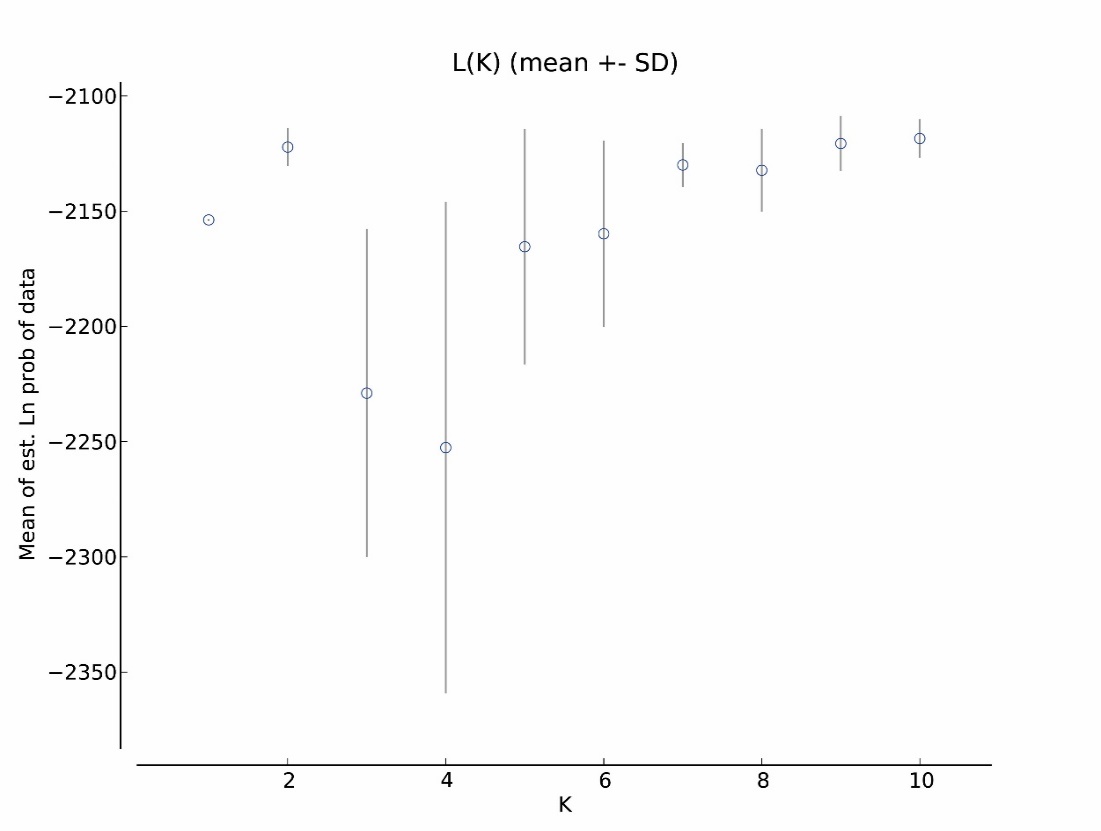


b)

a)

**Fig. S3**: The plot of a) the highest mean likelihood *L*(*K*) and b) Delta *K* using sampling location as *a priori.*


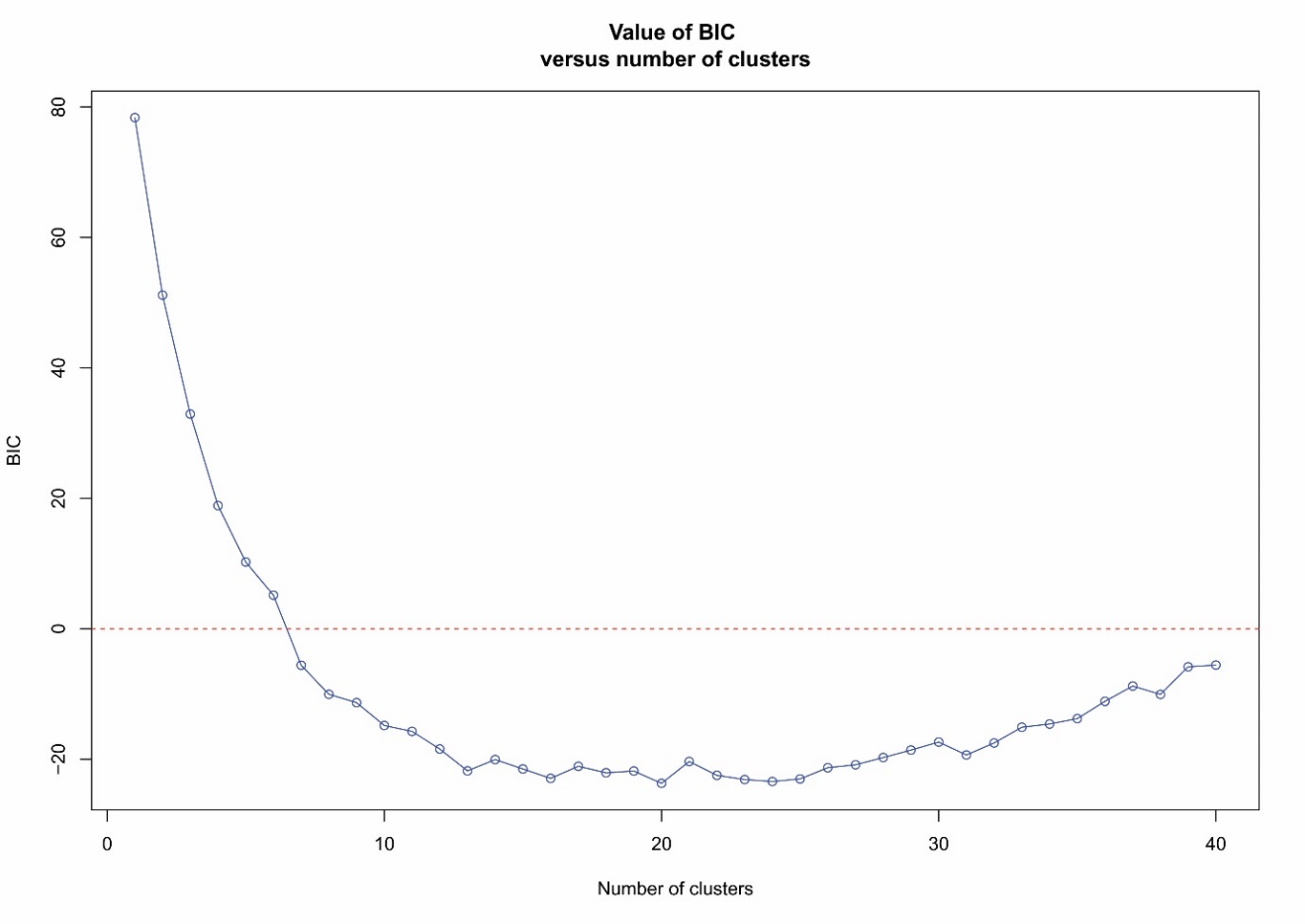


**Fig. S4:** The of the BIC against the number of clusters.

**References:**

1. Jogayya, K. N., Meganathan, P. R., Dubey, B. & Haque, I. Novel microsatellite DNA markers for Indian Gharial (*Gavialis gangeticus*). *Conservation Genetics Resources* **5**, 787–790 (2013).

2. Fitzsimmons, N. N. *et al.* Microsatellite markers for Crocodylus: new genetic tools for population genetics, mating system studies and forensics. in *Crocodilian Biology and Evolution* (eds. Grigg, G. C., Seebacher, F. & Franklin, C. E.) 51–57 (Surrey Beatty & Sons, Chipping Norton, 2000).

3. Aggarwal, R. K., Lalremruata, A. & Dubey, B. Development of fourteen novel microsatellite markers of *Crocodylus palustris*, the Indian mugger, and their cross-species transferability in ten other crocodilians. *Conservation Genetics Resources* **7**, 197–200 (2014).

4. Hinlo, M. R. P. *et al.* Population genetics implications for the conservation of the Philippine Crocodile *Crocodylus mindorensis* Schmidt, 1935 (Crocodylia: Crocodylidae). *Journal of Threatened Taxa* **6**, 5513–5533 (2014).

5. Miles, L. G., Isberg, S. R., Moran, C., Hagen, C. & Glenn, T. C. 253 Novel polymorphic microsatellites for the saltwater crocodile (*Crocodylus porosus*). *Conservation Genetics* **10**, 963–980 (2009).

6. Yu, D. *et al.* Analysis of genetic variation and bottleneck in a captive population of Siamese crocodile using novel microsatellite loci. *Conservation Genetics Resources* **3**, 217–220 (2011).

7. Glenn, T. C. *et al.* Characterization of Microsatellite DNA Loci in American Alligators. *Copeia* **3**, 591–601 (1998).

8. Davis, L. M. *et al.* Microsatellite DNA analyses support an east-west phylogeographic split of American alligator populations. *Journal of Experimental Zoology* **294**, 352–372 (2002).
